# Anterior Fontanelle Dermoid Cyst: Surgical Technique

**DOI:** 10.7759/cureus.16348

**Published:** 2021-07-12

**Authors:** Aurelio Ponce-Ayala, JP Navarro-Garcia de Llano, Javier Degollado-Garcia, Nickjail Hernández-Álvarez, Rafael Mendizabal-Guerra

**Affiliations:** 1 Neurosurgery, Hospital Juarez De Mexico, Mexico City, MEX; 2 Neurocirugía Vascular, Instituto Nacional de Neurología y Neurocirugía, Mexico City, MEX; 3 Neurosurgery, Hospital Juarez de Mexico, Mexico City, MEX

**Keywords:** anterior fontanelle, congenital dermoid cyst, inclusion cyst, scalp, dermoid cysts, congenital epidermoid cyst

## Abstract

Dermoid cysts are benign congenital lesions that usually appear on the surface of the skull, mainly on the anterior fontanelle. Diagnosis is usually made in the first months of life by physical examination and imaging studies such as CT, MRI, or ultra sound (US) Doppler. It is important to distinguish it from other similar lesions that represent greater surgical complexity, morbidity, and mortality. In this work, we show the principle differential diagnoses, the diagnostic approach, and the surgical technique used in the resection of the dermoid cyst located over the anterior fontanelle.

## Introduction

Congenital dermoid cysts (CDCs) are benign lesions that originate from the entrapment of the surface ectoderm along the lines of embryonic fusion [1]. They usually present in the first few months of life and then gradually enlarge due to internal desquamation and ultimately become symptomatic as a result of aggrandizement, rupture, and even, in some cases, extension into surrounding structures [2-4]. Cysts can be either dermoid or epidermoid and are classified mainly by their histological characteristics [5,6]. Dermoid cysts are rare lesions, with an incidence of 0.1-0.5% of cranial tumors located in the midline; 25% of these cysts are located in the anterior fontanel [7]. Fontanelles make up six areas of dense connective tissue that correspond to the union of two or more sutures and are made up of three layers: external (periosteum), middle (connective tissue), and internal (dura mater) [8].

The most common differential diagnosis of these lesions, described in the literature, are epidermoid cyst, encephalocele, lipoma, cephalohematoma, hemangioma, and sinus pericranii [9]. MRI, CT, and/or Doppler ultrasound (US) imaging is important to distinguish between differentials, and for surgical planning [2,6]. Surgical resection should be performed for aesthetic reasons [6]. Here we show the case of a three-month-old patient who presented a lump in the anterior fontanel from the first month of life.

## Case presentation

**Figure 1:**
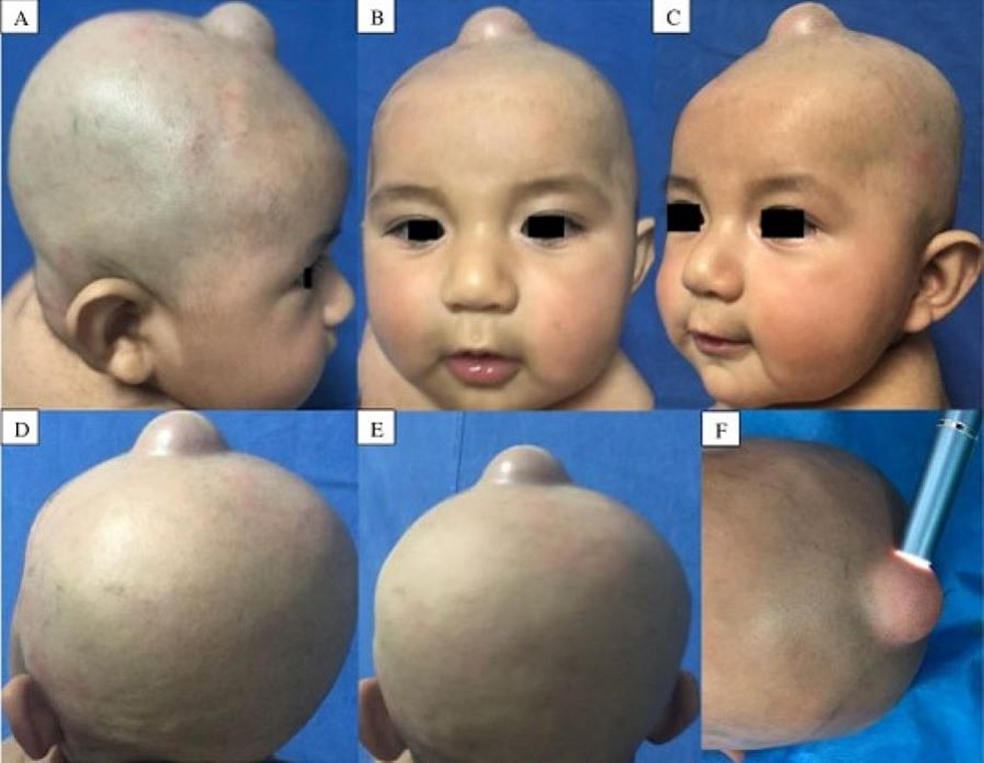
Patient’s physical examination when first brought to us. We appreciate a rubbery, nonpulsatile, nontender, skin colored lesion, located in the anterior fontanelle. A) Right lateral view B) Anterior view C) Left lateral view D, E) Posterior view F) Transillumination test

A three-month-old infant with no significant past medical history presented with a subcutaneous soft mass over the anterior fontanelle noted from the first month of life. The patient was born without complications and has no relevant prenatal history. Physical examination showed a well-defined mass of 3 cm diameter, covered by undamaged skin located in anterior fontanelle, soft consistency, barely depressible, with no changes in overlying skin coloration, and minimum transillumination was appreciated. The lesion was neither pulsating nor painful (Figure 1).

**Figure 2:**
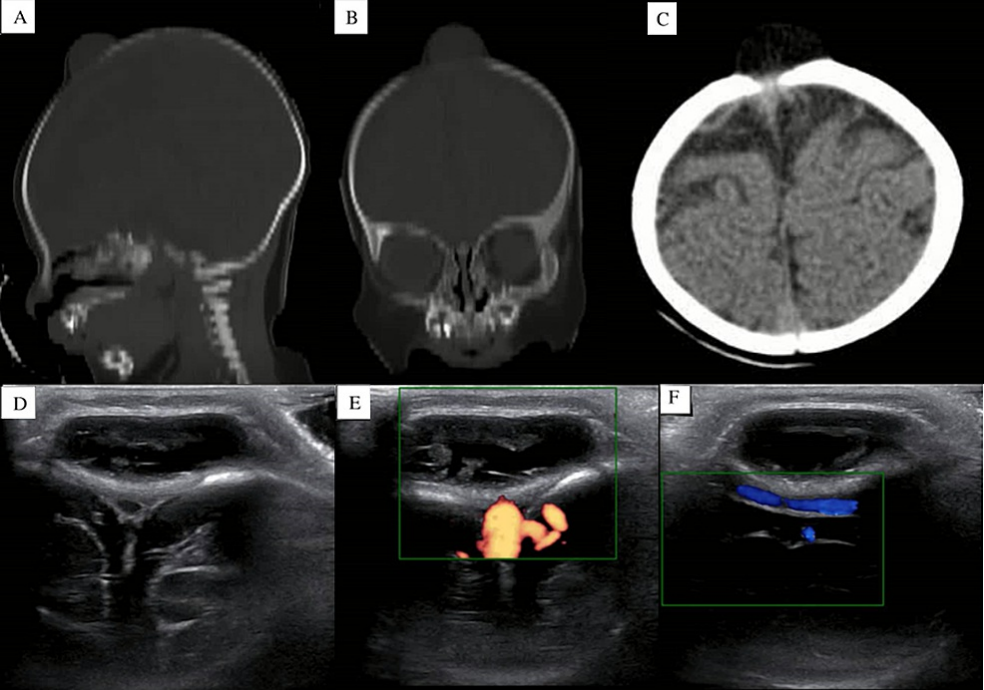
CT and Doppler US A) Head CT from a sagittal view. B) Head CT from a coronal view. C) Head CT from an axial view. In all of them, we can appreciate an extracranial mass, consistent with an anterior fontanelle dermoid cyst. D) Ultrasound from a coronal view. E) Doppler Ultrasound from a coronal view. F) Doppler Ultrasound from a longitudinal view. In neither of them, we see blood flow inside the cyst.

On neurological examination, he did not present delay for age or alterations in neurological development. Ultrasonography showed a subcutaneous hypoechogenic cystic lesion without vascularization. CT scan confirmed the localization of the lesion above the anterior fontanelle and the absence of involvement of underlying encephalic structures (Figure 2).

**Figure 3:**
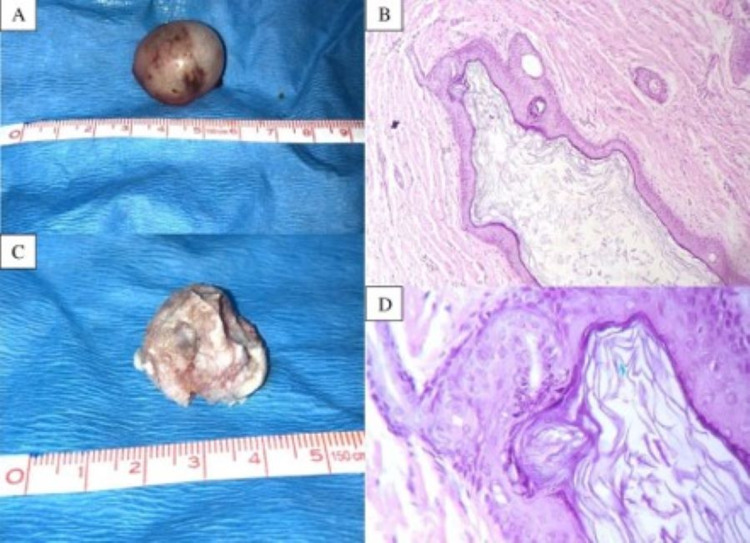
Macro and micro pathology of the dermoid cyst after excision A) 3 cm dermoid cyst before drainage. B) Dermoid cyst after drainage. C, D) Histology

Findings were suggestive of a congenital inclusion cyst of the anterior fontanelle, and surgical removal was achieved without complications. The histological analysis showed the presence of keratin, hair follicles, sweat, and sebaceous glands delimited by a layer of stratified squamous epithelium (Figure 3). Also, the fluid inside the cyst was basically composed of glucose and protein (Table 1). 

**Table 1:** Cyst's fluid test

PARAMETER	RESULT
Glucose	3 mg/dl
Protein	4.1 mg/dl
Appearance	Colorless/Liquid
Blood	Negative
Cell Count	Negative

Surgical technique

**Figure 4:**
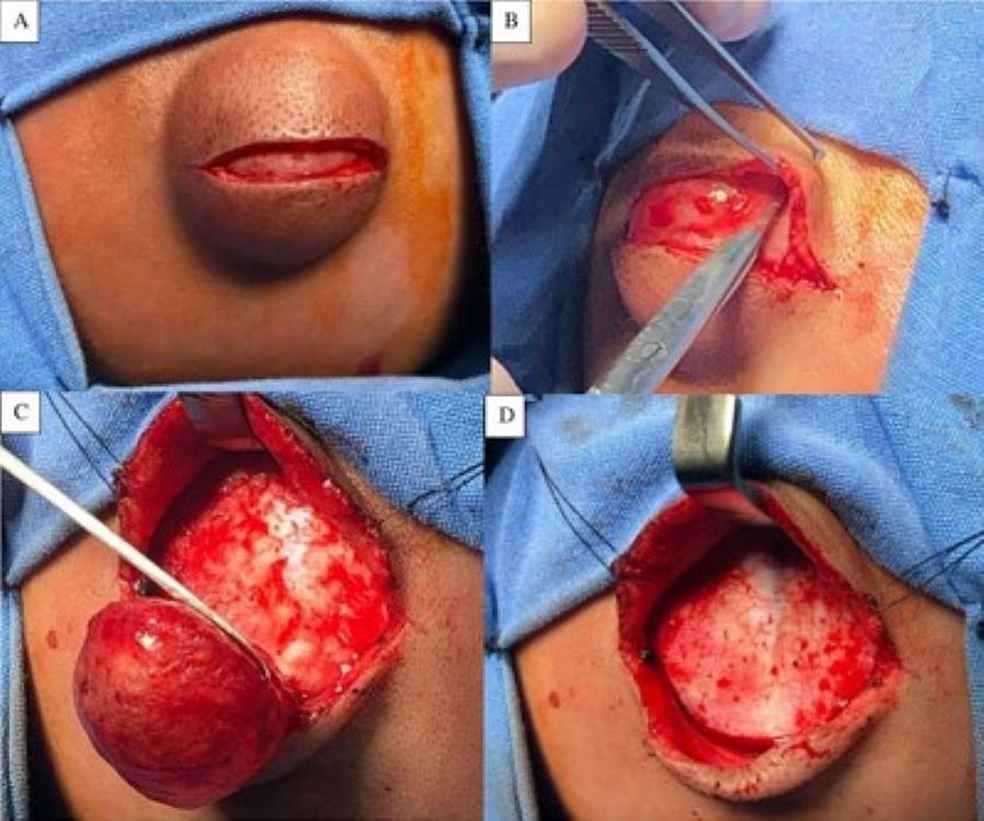
Surgical procedure A) Skin incision. B) Releasing of the cyst's borders. C) Tissue being removed from the periosteum layer. D) Periosteum after mass was excised.

**Figure 5:**
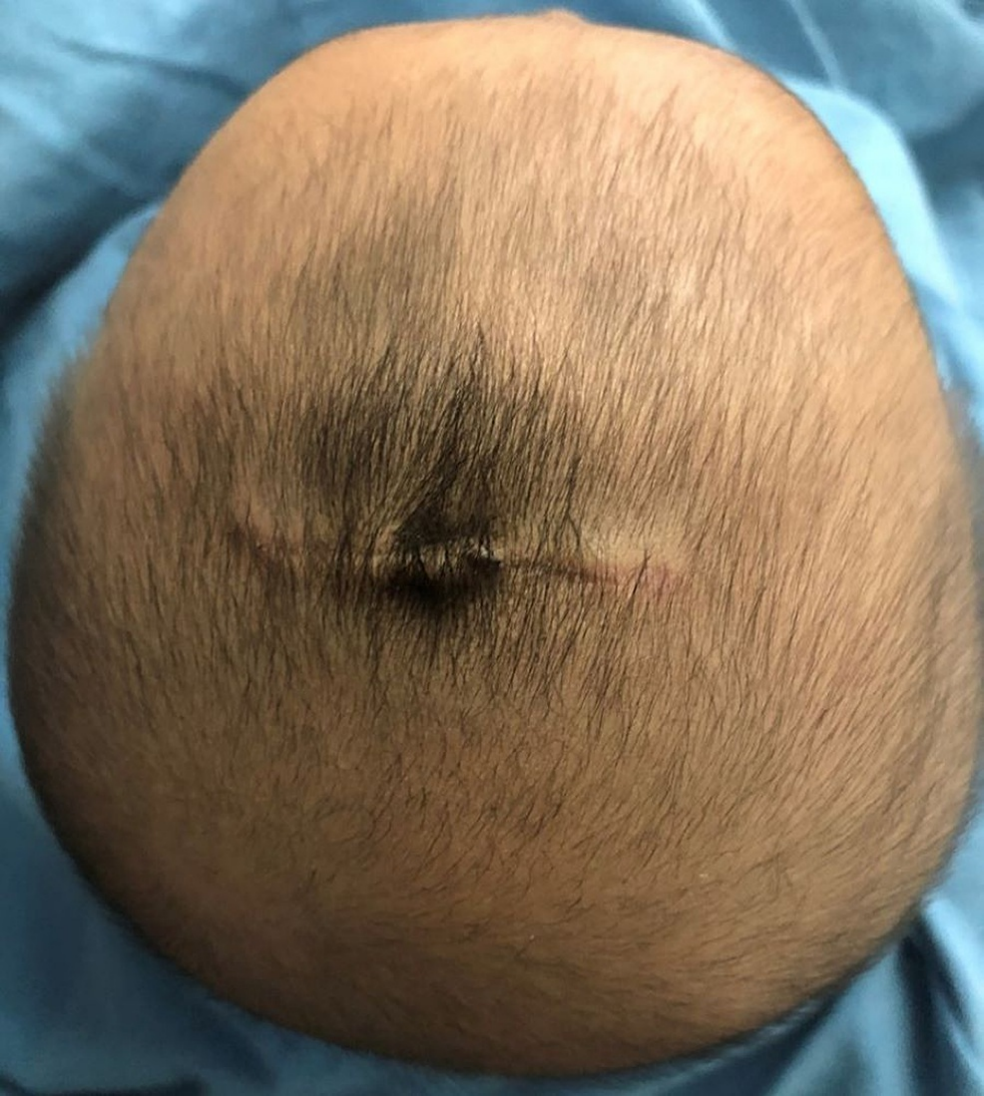
Incision site two months after surgery

We made a coronal-oriented linear incision on the cyst, including skin, subcutaneous cellular tissue, and aponeurotic galea (Figure 4A). At the subgaleal level, through traction of the skin flap, we expose and cut with scissors areolar tissue filaments adhered to the cyst, releasing its dorsal, anterior, posterior, and both lateral surfaces (Figure 4B). As the last step, due to a possible injury to the anterior fontanelle’s layers (periosteum, connective tissue, and dura mater) or the superior sagittal sinus, we completed a sharp dissection of the ventral surface with the same technique, avoiding any damage to the periosteum (Figure 4C, 4D). Once hemostasis is achieved, we proceed to closure the aponeurotic galea using continuous vicryl 4-0 suture and skin with 4-0 subdermal nylon. We show an image of the surgical site two months after surgery (Figure 5).

## Discussion

In this paper, as well as describing the clinical case and the surgical technique of a patient with a dermoid cyst of the anterior fontanelle, we mention the diagnostic importance of these types of malformations, and its wide range of alternative entities, since some lesions may be simple to handle and do not endanger the life of the patient, and others, like sinus pericranii and encephalocele, do become more complex. We present a table showing the differential diagnoses of a mass at the level of the anterior fontanelle (Table 2).

**Table 2:** Differential diagnoses of an anterior fontanelle mass CT: Computed tomography, MRI: Magnetic Resonance Image, US: Ultrasound

LESION	DEFINITION	CLINICAL CHARACTERISTICS	DIAGNOSIS	TREATMENT
Dermoid Cyst [4]	Congenital subcutaneous lesions that are usually distributed along embryonic fusion lines	Slow-growing, asymptomatic, rubbery, non-pulsatile, non-compressible, non-tender, skin-colored	CT, MRI, Doppler US	Surgical excision
Sinus pericranii [10]	Rare vascular malformation between extracranial vessels and the superior longitudinal sinus	Blue-purple subcutaneous nodule, compressible, non-tender, increases its size with Valsalva maneuver	CT, MRI, Doppler US	Surgical repair
Epidermoid Cyst [11]	Epidermal cells located within the dermis proliferate and do not communicate with the surface	Similar to dermoid cyst, but usually develop after puberty	CT, MRI, Doppler US	Surgical excision
Cephalohematoma [12]	Subperiosteal collection of blood caused by rupture of vessels	Swelling that does not cross suture lines, may or may not be accompanied by discoloration	CT, MRI, US	Majority resolve spontaneously
Encephalocele [13]	Malformation where calvarial and dural defects allow extracranial herniation of leptomeninges, brain or CSF	May present with marked craniofacial deformities (hypertelorism, telecanthus, orbital dystopia, or unilateral micro/anophthalmos)	Prenatal US	Surgical repair
Haemangioma [14]	Most common vascular tumors, characterized by a growth phase and an involution phase	Patch with telangiectasias with surrounding pallor. Subtle and solitary lesions	Clinical, US, CT, MRI	Self-limited
Lipoma [15]	Most common benign soft-tissue neoplasms, mature fat cells enclosed by thin fibrous capsules	Soft, painless subcutaneous nodules ranging from 1 to >10 cm.	Clinical, US	Surgical excision

This differentiation can be carried out through imaging studies such as CT or MRI, which would confirm the extracranial position [9]. On CT, the dermoid cyst is observed as a cystic well-defined mass, with an attenuation coefficient similar to soft tissues, and on occasions, we can appreciate the separation between the intra and extracranial structures. On the other hand, the MRI may show a homogenous lesion, hypointense on T1-weighted, and hyperintense on T2-weighted images, without vascular structures [6,7,9]. In this case, Doppler ultrasound is also highly useful, which allows us to verify the absence of blood flow in the lesion, in a more accessible way and thus proceed in a safer way to the surgical procedure.

The definitive diagnosis of these types of lesions is made with histologic study, in which we can observe the presence of hair follicles, sweat glands, and sebaceous glands [9].

The surgical management of this type of lesion is mainly due to aesthetic reasons, however, we describe the surgical technique that, although it may seem simple, there are structures (e.g., superior longitudinal sinus) that we must take into account to avoid a poor outcome in our patient [6].

These lesions have an excellent prognosis and the chance for recurrence is rare [6].

## Conclusions

Early and precise diagnosis of these types of lesions is very relevant to distinguish them from complex diseases that can become more challenging for us to deal with in the operating room. The use of imaging tools such as CT, MRI, and/or Doppler US, is essential when making the presumptive diagnosis of these types of cysts, so we can ensure an appropriate surgical approach, to preserve vital structures in our patients. We encourage all healthcare physicians to use the technology available in their health centers, so the wide range of differential diagnoses can be reduced to those that do not endanger the patient's life. The surgical technique exposed in this paper can be performed at any standard operating room and does not require the use of advanced tools.
